# Supplementary material for: A Literature Review of Modeling Approaches Applied to Data Collected in Automatic Milking Systems
Source: Animals (Basel). 2023 Jun 8;13(12):1916. doi: 10.3390/ani13121916 (PMC10294954; doi:10.3390/ani13121916)
Supplement: Supplementary file 1 [file animals-13-01916-s001.zip › animals-2423549-supplementary/Table_S1.pdf]

Table S1. Summary of reviewed articles.

**Application domain**	**Problem**	**Model**	**Data sets**	**Variables**	**References**
Health					
	udder health status	GLM	322 cows with 549 lactations	EC	Norberg et al. (2004) [49]
	clinical mastitis	Fuzzy logic	200 cows	EC, SCC	Kamphuis et al. (2008a) [50]
	assessment of use of SCC at quarter level	detection algorithms	3,191 quarter milkings	EC, SCC	Mollenhorst et al. (2010) [51]
	clinical mastitis	detection algorithms	52 cows	EC, SCC	Khatun et al. (2017) [52]
	mastitis and its stage of progression	MLP and SOM	48 546 milking records from 194 cows	EC, quarter milk yield	Sun et al. (2009) [53]
	clinical mastitis	detection algorithms	eight dairy herds (283 cows in total)	EC, milk color	Hovinen et al. (2006) [54]
	subclinical mastitis	Logistic regression model	204 cows	SCC, EC, milking day, milk colors, lactation order, pH, freezing point	Altay et al. (2019) [56]
	mastitis	dynamic deterministic model	The model functionality was investigated using simulated data, and real-farm data (100 cows and 76,257 records from every milking)	LDH, days from calving, breed, parity, milk yield, udder characteristics, other disease records, electrical conductivity, and herd characteristics.	Chagunda et al. (2006) [57]
	mastitis	NNs, GAMs	401 cows, 664 lactations	EC, LDH, SCC, milk yield	Ankinakatte et al. (2013) [59]
	mastitis	RNN	89 dairy farms (8,152 cows)	milk traits, behavioral characteristics, cow traits,	Naqvi et al. (2022a) [63]

				environmental/farm-level characteristics and daily variances	
	clinical mastitis	Logistic regression model	1,549 cows	peak milk flow rate, parity, quarter position, day in milk at diagnosis of clinical mastitis, udder milk yield, and milking interval.	Penry et al. (2017) [62]
	mastitis alert	Time-series detection models	111 cows	milk yield and EC	de Mol and Ouweltjes (2001) [67]
	mastitis alert	Fuzzy logic	25 cows, 29,033 milking records	EC	De Mol and Woldt (2001) [68]
	mastitis alert	DNN	1,900 cows	EC, milk yield, milking interval, milking duration	Khamaysa Hajaya et al. (2019) [69]
	clinical mastitis alert	Naïve BN	602 cows, 511,744 milking records	AMS information and prior cow information (parity, days in milk, season of the year, somatic cell count history and clinical mastitis history)	Steeneveld et al. (2010a) [44]
	clinical mastitis alert	Naïve BN	22,860 cows, 28,137 lactations	AMS information and prior cow information (parity, days in milk, season of the year, somatic cell count history and clinical mastitis history)	Steeneveld et al. (2010b) [70]
	clinical mastitis alert	RF	1,109 cows	EC, milk production, dead milking time, and milk flow	Kamphuis et al. (2010a; 2010b) [72, 71]
	mastitis DOI	dynamic deterministic model (same of Chagunda et al., 2006)	496,014 milking records	SCC, LDH	Friggens et al. (2007) [58]

	mastitis DOI	dynamic deterministic model	332 cows	EC, SCC, LDH	Højsgaard and Friggens (2010) [74]
	clinical mastitis risk	algorithm developed by the authors	1,938 cows, 595,927 milking records	online cell count	Sørensen et al. (2016) [75]
	mastitis	fuzzy logic	478 cows, 403,537 milking records	EC, milk yield, and milk flow rate.	Cavero et al. (2006) [46]
	mastitis	NN		EC, milk yield, milk flow and days in milk.	Cavero et al. (2008) [76]
	mastitis	NN		EC, milk production rate, milk flow rate and days in milk.	Krieter et al. (2007) [77]
	clinical and subclinical mastitis	SVM, NN, ANFIS, Fuzzy logic	170 cows, 346 milking records	current lactation number, milk yield, EC, average milking duration and season	Mammadova and Keskin (2013, 2015a, 2015b) [78-80]
	cow composite SCC	GAM, RF, and MLP	372 cows, 30,734 milking records	87 variables	Anglart et al. (2020) [81]
	subclinical mastitis	NN, Naïve Bayes, GLM, Logistic Regression, DT, Gradient-Boosted Tree (GBT) and RF	364,249 milking records	milk volume, lactose concentration, EC, protein concentration, peak flow and milking time	Ebrahimi et al. (2019) [82]
	mastitis	Linear Discriminant Analysis (LDA), GLM with logit link function, Naïve Bayes, Classification and Regression Trees, k-NN, SVM, RF and NN	791 herds, 14,064 cows, 18,442 milking records	information on herd, cows (ID, breed, stage of lactation and parity), date of sample collection, daily milk production, milk composition	Bobbo et al. (2021) [47]
	clinical mastitis	k-NN RF, SVM, and AdaBoost	60 cows	milk production, EC, milk flow, pH, milk temperature	Tian et al. (2020) [83]
	mastitis risk	26 classification models	6,600 cows	15 variables	Ghafoor and Sitkowska (2021)

					[84]
	mastitis pathogens	unsupervised (USNN) and supervised neural network (SNN)	4,852 quarter milk samples	SCC, electrical resistance, fat percentage, protein percentage, and bacteriology records	Hassan et al. (2009) [86]
	mastitis pathogens	BN	274 dairy herds (mean herd size of 75 cows)	parity, month in lactation, location of infected quarter, season SCC, clinical mastitis history, pathogen history, milk color, texture of the milk	Steeneveld et al. (2009) [88]
	mastitis pathogens	DT	9 herds, 772 cows	EC, milk color, and milk yield	Kamphuis et al. (2011) [85]
	mastitis pathogens	Detection algorithm	10 herds, 852 cows	AMS mastitis alerts.	Castro et al. (2015) [89]
	health status	fuzzy logic	147 cows	milk production, milk flow, EC, and activity	Liberati and Zappavigna (2009) [90]
	post-calving diseases	DT	250 cows	milk yield, milking duration, rumination, activity, concentrate feed intake and visits to the milking robot	Steensels et al. (2016) [91]
	Clinical mastitis	Generalized linear mixed models (GLMMs)	7096 cows	AMS information and prior cow information	Bausewein et al. (2022) [73]
	Chronic mastitis	Gradient-boosting trees	herd sizes of lactating cows ranging from 55 to 638 cows	SCC, EC, blood in the milk, parity, time interval between milkings, milk yield days in milk.	Bonestroo et al. (2022) [65]
	mastitis	RNNs	Simulated data	Rumination data, animal activity,	Naqvi et al. (2022b) [64]

				SCC, parity, days in milk, milk yield	
	Health disorders	k-NN RF, SVM, AdaBoost, Naïve Bayes, DT	280 cows	Season, days in milk, milk yield, parity, activity, rumination time, EC	Zhou et al. (2022) [92]
Production					
	milk production per milking	Dynamic linear modeling (DLM)	169,774 milkings	variables from AMS (milk yield and SCC)	Jensen et al. (2018) [98]
	milk yield	DT	3778 cows	daily milk yield of cows, daily milking frequency, milking speed, and the number of milked quarters	Piwczyński et al. (2020) [101]
	milk yield per lactation	Classification and Regression Trees (CART) algorithm	524 cows, 18,055 milking records	rumination, milking parameters, colostrum/milk traits	Klis et al. (2021a, 2021b) [102, 104]
	milk yield	RF	91 cows	environmental parameters, day in the lactation curve	Bovo et al., 2021 [18]
	milk yield, fat and protein content, and actual cow concentrate feed intake.	NN	781 cows	programmed concentrate feed and weight combined with microclimatic parameters	Fuentes et al. (2020) [106]
	Milk yield, milk composition and milk frequency	XGBOOST algorithm	80 cows	Environmental, productivity, health and behavior variables	Ji et al. (2022) [107]
Cows Behavior and herd management					

	physical activity	Cluster Analysis (Ward's method and Kohonen's networks)	10 cows	activity, lactation phases, daylight duration, temperature, relative humidity	Adamczyk et al. (2017) [94]
	social interactions	SVM, CNNs	252 cows	correct position of cows and distances from every pair of cows	Guzhva et al. (2016; 2018) [96, 97]
	herd management	k-means clustering	65 cows	production and behavioral features	Bonora et al. (2018) [8]
