# Supplementary material for: A Literature Review of Modeling Approaches Applied to Data Collected in Automatic Milking Systems
Source: Animals (Basel). 2023 Jun 8;13(12):1916. doi: 10.3390/ani13121916 (PMC10294954; doi:10.3390/ani13121916)
Supplement: Supplementary file 1 [file animals-13-01916-s001.zip › animals-2423549-supplementary/Table_S2.pdf]

Table S2. List of abbreviations

Automatic milking systems	AMS
Machine Learning	ML
Precision Livestock Farming	PLF
True Positives	TP
True Negatives	TN
False Positives	FP
False Negatives	FN
Receiver Operating Characteristic	ROC
Area Under the ROC	AUR
Mean Absolute Error	MAE
Root Mean Squared Error	RMSE
International Standards Organization	ISO
Electrical Conductivity	EC
Somatic Cell Count	SCC
Differential Somatic Cell Count	DSCC
Generalized Linear Models	GLM
Generalized Additive Models	GAM
Decision Tree	DT
Random Forest	RF
k-Nearest Neighbors	k-NN
Support Vector Machine	SVM
Bayesian Network	BN
Neural Networks	NN
Multilayer Perceptron	MLP
Back Propagation Neural Network	BPNN
Probabilistic Neural Network	PNN
Recurrent Neural Network	RNN

Convolutional Neural Network	CNN
Self-Organizing Maps	SOM
Hierarchical clustering	HC
Adaptive Neuro Fuzzy Inference System	ANFIS
Genetic Algorithms	GA
Lactate Dehydrogenase	LDH
Degree of Infection	DOI
Elevated Mastitis Risk	EMR
Temperature Humidity Index	THI
