# Supplementary material for: A Literature Review of Modeling Approaches Applied to Data Collected in Automatic Milking Systems
Source: Animals (Basel). 2023 Jun 8;13(12):1916. doi: 10.3390/ani13121916 (PMC10294954; doi:10.3390/ani13121916)
Supplement: Supplementary file 1 [file animals-13-01916-s001.zip › Supplementary_material/Table_S1.docx]

**Table S1**. Summary of reviewed articles.

| **Application domain** | **Problem** | **Model** | **Data sets** | **Variables** | **References** |
| --- | --- | --- | --- | --- | --- |
| *Health* | | | | | |
|  | udder health status | GLM | 322 cows with 549 lactations | EC | Norberg et al. (2004) [49] |
|  | clinical mastitis | Fuzzy logic | 200 cows | EC, SCC | Kamphuis et al. (2008a) [50] |
|  | assessment of use of SCC at quarter level | detection algorithms | 3,191 quarter milkings | EC, SCC | Mollenhorst et al. (2010) [51] |
|  | clinical mastitis | detection algorithms | 52 cows | EC, SCC | Khatun et al. (2017) [52] |
|  | mastitis and its stage of progression | MLP and SOM | 48 546 milking records from 194 cows | EC, quarter milk yield | Sun et al. (2009) [53] |
|  | clinical mastitis | detection algorithms | eight dairy herds (283 cows in total) | EC, milk color | Hovinen et al. (2006) [54] |
|  | subclinical mastitis | Logistic regression model | 204 cows | SCC, EC, milking day, milk colors, lactation order, pH, freezing point | Altay et al. (2019) [56] |
|  | mastitis | dynamic deterministic model | The model functionality was investigated using simulated data, and real-farm data (100 cows and 76,257 records from every milking) | LDH, days from calving, breed, parity, milk yield, udder characteristics, other disease records, electrical conductivity, and herd characteristics. | Chagunda et al. (2006) [57] |
|  | mastitis | NNs, GAMs | 401 cows, 664 lactations | EC, LDH, SCC, milk yield | Ankinakatte et al. (2013) [59] |
|  | mastitis | RNN | 89 dairy farms (8,152 cows) | milk traits, behavioral characteristics, cow traits, environmental/farm-level characteristics and daily variances | Naqvi et al. (2022a) [63] |
|  | clinical mastitis | Logistic regression model | 1,549 cows | peak milk flow rate, parity, quarter position, day in milk at diagnosis of clinical mastitis, udder milk yield, and milking interval. | Penry et al. (2017) [62] |
|  | mastitis alert | Time-series detection models | 111 cows | milk yield and EC | de Mol and Ouweltjes (2001) [67] |
|  | mastitis alert | Fuzzy logic | 25 cows, 29,033 milking records | EC | De Mol and Woldt (2001) [68] |
|  | mastitis alert | DNN | 1,900 cows | EC, milk yield, milking interval, milking duration | Khamaysa Hajaya et al. (2019) [69] |
|  | clinical mastitis alert | Naïve BN | 602 cows, 511,744 milking records | AMS information and prior cow information (parity, days in milk, season of the year, somatic cell count history and clinical mastitis history) | Steeneveld et al. (2010a) [44] |
|  | clinical mastitis alert | Naïve BN | 22,860 cows, 28,137 lactations | AMS information and prior cow information (parity, days in milk, season of the year, somatic cell count history and clinical mastitis history) | Steeneveld et al. (2010b) [70] |
|  | clinical mastitis alert | RF | 1,109 cows | EC, milk production, dead milking time, and milk flow | Kamphuis et al. (2010a; 2010b) [72, 71] |
|  | mastitis DOI | dynamic deterministic model (same of Chagunda et al., 2006) | 496,014 milking records | SCC, LDH | Friggens et al. (2007) [58] |
|  | mastitis DOI | dynamic deterministic model | 332 cows | EC, SCC, LDH | Højsgaard and Friggens (2010) [74] |
|  | clinical mastitis risk | algorithm developed by the authors | 1,938 cows, 595,927 milking records | online cell count | Sørensen et al. (2016) [75] |
|  | mastitis | fuzzy logic | 478 cows, 403,537 milking records | EC, milk yield, and milk flow rate. | Cavero et al. (2006) [46] |
|  | mastitis | NN |  | EC, milk yield, milk flow and days in milk. | Cavero et al. (2008) [76] |
|  | mastitis | NN |  | EC, milk production rate, milk flow rate and days in milk. | Krieter et al. (2007) [77] |
|  | clinical and subclinical mastitis | SVM, NN, ANFIS, Fuzzy logic | 170 cows, 346 milking records | current lactation number, milk yield, EC, average milking duration and season | Mammadova and Keskin (2013, 2015a, 2015b) [78-80] |
|  | cow composite SCC | GAM, RF, and MLP | 372 cows, 30,734 milking records | 87 variables | Anglart et al. (2020) [81] |
|  | subclinical mastitis | NN, Naïve Bayes, GLM, Logistic Regression, DT, Gradient-Boosted Tree (GBT) and RF | 364,249 milking records | milk volume, lactose concentration, EC, protein concentration, peak flow and milking time | Ebrahimi et al. (2019) [82] |
|  | mastitis | Linear Discriminant Analysis (LDA), GLM with logit link function, Naïve Bayes, Classification and Regression Trees, k-NN, SVM, RF and NN | 791 herds, 14,064 cows, 18,442 milking records | information on herd, cows (ID, breed, stage of lactation and parity), date of sample collection, daily milk production, milk composition | Bobbo et al. (2021) [47] |
|  | clinical mastitis | k-NN RF, SVM, and AdaBoost | 60 cows | milk production, EC, milk flow, pH, milk temperature | Tian et al. (2020) [83] |
|  | mastitis risk | 26 classification models | 6,600 cows | 15 variables | Ghafoor and Sitkowska (2021) [84] |
|  | mastitis pathogens | unsupervised (USNN) and supervised neural network (SNN) | 4,852 quarter milk samples | SCC, electrical resistance, fat percentage, protein percentage, and bacteriology records | Hassan et al. (2009) [86] |
|  | mastitis pathogens | BN | 274 dairy herds (mean herd size of 75 cows) | parity, month in lactation, location of infected quarter, season SCC, clinical mastitis history, pathogen history, milk color, texture of the milk | Steeneveld et al. (2009) [88] |
|  | mastitis pathogens | DT | 9 herds, 772 cows | EC, milk color, and milk yield | Kamphuis et al. (2011) [85] |
|  | mastitis pathogens | Detection algorithm | 10 herds, 852 cows | AMS mastitis alerts. | Castro et al. (2015) [89] |
|  | health status | fuzzy logic | 147 cows | milk production, milk flow, EC, and activity | Liberati and Zappavigna (2009) [90] |
|  | post-calving diseases | DT | 250 cows | milk yield, milking duration, rumination, activity, concentrate feed intake and visits to the milking robot | Steensels et al. (2016) [91] |
|  | Clinical mastitis | Generalized linear mixed models (GLMMs) | 7096 cows | AMS information and prior cow information | Bausewein et al. (2022) [73] |
|  | Chronic mastitis | Gradient-boosting trees | herd sizes of lactating cows ranging from 55 to 638 cows | SCC, EC, blood in the milk, parity, time interval between milkings, milk yield  days in milk. | Bonestroo et al. (2022) [65] |
|  | mastitis | RNNs | Simulated data | Rumination data, animal activity, SCC, parity, days in milk, milk yield | Naqvi et al. (2022b) [64] |
|  | Health disorders | k-NN RF, SVM, AdaBoost, Naïve Bayes, DT | 280 cows | Season, days in milk, milk yield, parity, activity, rumination time, EC | Zhou et al. (2022) [92] |
| *Production* | | | | | |
|  | milk production per milking | Dynamic linear modeling (DLM) | 169,774 milkings | variables from AMS (milk yield and SCC) | Jensen et al. (2018) [98] |
|  | milk yield | DT | 3778 cows | daily milk yield of cows, daily milking frequency, milking speed, and the number of milked quarters | Piwczyński et al. (2020) [101] |
|  | milk yield per lactation | Classification and Regression Trees (CART) algorithm | 524 cows, 18,055 milking records | rumination, milking parameters, colostrum/milk traits | Klis et al. (2021a, 2021b) [102, 104] |
|  | milk yield | RF | 91 cows | environmental parameters, day in the lactation curve | Bovo et al., 2021 [18] |
|  | milk yield, fat and protein content, and actual cow concentrate feed intake. | NN | 781 cows | programmed concentrate feed and weight combined with microclimatic parameters | Fuentes et al. (2020) [106] |
|  | Milk yield, milk composition and milk frequency | XGBOOST algorithm | 80 cows | Environmental, productivity, health and behavior variables | Ji et al. (2022) [107] |
| *Cows Behavior and herd management* | | | | | |
|  | physical activity | Cluster Analysis (Ward’s method and Kohonen’s networks) | 10 cows | activity, lactation phases, daylight duration, temperature, relative humidity | Adamczyk et al. (2017) [94] |
|  | social interactions | SVM, CNNs | 252 cows | correct position of cows and distances from every pair of cows | Guzhva et al. (2016; 2018) [96, 97] |
|  | herd management | k-means clustering | 65 cows | production and behavioral features | Bonora et al. (2018) [8] |
