# Supplementary material for: A Literature Review of Modeling Approaches Applied to Data Collected in Automatic Milking Systems
Source: Animals (Basel). 2023 Jun 8;13(12):1916. doi: 10.3390/ani13121916 (PMC10294954; doi:10.3390/ani13121916)
Supplement: Supplementary file 1 [file animals-13-01916-s001.zip › Supplementary_material/Table_S2.docx]

Table S2. List of abbreviations

| Automatic milking systems | AMS |
| --- | --- |
| Machine Learning | ML |
| Precision Livestock Farming | PLF |
| True Positives | TP |
| True Negatives | TN |
| False Positives | FP |
| False Negatives | FN |
| Receiver Operating Characteristic | ROC |
| Area Under the ROC | AUR |
| Mean Absolute Error | MAE |
| Root Mean Squared Error | RMSE |
| International Standards Organization | ISO |
| Electrical Conductivity | EC |
| Somatic Cell Count | SCC |
| Differential Somatic Cell Count | DSCC |
| Generalized Linear Models | GLM |
| Generalized Additive Models | GAM |
| Decision Tree | DT |
| Random Forest | RF |
| k-Nearest Neighbors | k-NN |
| Support Vector Machine | SVM |
| Bayesian Network | BN |
| Neural Networks | NN |
| Multilayer Perceptron | MLP |
| Back Propagation Neural Network | BPNN |
| Probabilistic Neural Network | PNN |
| Recurrent Neural Network | RNN |
| Convolutional Neural Network | CNN |
| Self-Organizing Maps | SOM |
| Hierarchical clustering | HC |
| Adaptive Neuro Fuzzy Inference System | ANFIS |
| Genetic Algorithms | GA |
| Lactate Dehydrogenase | LDH |
| Degree of Infection | DOI |
| Elevated Mastitis Risk | EMR |
| Temperature Humidity Index | THI |
